# A large thyroglossal duct cyst and its management: a case report

**DOI:** 10.11604/pamj.2023.44.10.35448

**Published:** 2023-01-05

**Authors:** Athanasios Luca Fountarlis, Petros Koltsidopoulos, Jiannis Hajiioannou, Vasileios Lachanas, Nick Kalogritsas, Efthymios Solomi, Charalampos Skoulakis

**Affiliations:** 1 Department of Otorhinolaryngology, University Hospital of Larisa, Larisa, Greece

**Keywords:** Thyroglossal duct cyst, Sistrunk’s procedure, complications, aspiration biopsy, case report

## Abstract

Thyroglossal duct cysts (TGDC) are congenital neck cysts, formed as a result of the failure of the thyroglossal duct to involute during embryogenesis and their mean size is 1.5-2.4 cm. We present a case of a 44-year-old male who presented with a history of a large anterior neck mass measuring 8.7x6x6.4 cm and causing dysphagia and mild dyspnea. After being mistaken for a goiter, a clinical diagnosis of TGDC was made based on history, clinical and radiographic findings. The patient was treated with Sistrunk’s procedure. No recurrence was noted on follow-up. Thyroglossal duct cysts are generally well-defined small lesions, but even bigger ones are not linked with severe symptomatology. The larger size at presentation may increase the list of potential diagnoses and lead to diagnostic dilemmas. Every effort should be made to rule out malignancy before surgery. Sistrunk’s procedure with dissection of the posterior hyoid space should be the standard of care.

## Introduction

The thyroid gland is an endocrine gland that begins to develop in the fourth week of gestation from the endodermis layer of the developing floor of the pharynx. It descends caudally from the foramen cecum and reaches its location below the thyroid cartilage by the end of the seventh week [1,2]. The thyroglossal duct is an epithelial remnant that connects the thyroid gland to the foramen cecum and normally involutes by the tenth week of gestation. Failure of the thyroglossal duct to involute predisposes to the formation of cysts, sinuses, or fistulas, anywhere along its pathway [3-5]. A thyroglossal duct cyst (TGDC) is usually located close to and inferior to the hyoid bone, although intralingual, substernal, and mediastinal locations have been reported [6,7]. It presents as a painless neck mass in the midline or in a slightly lateral position and moves upwards during deglutition or tongue protrusion [8]. Sometimes, during an upper respiratory tract infection, the TGDC may get infected and become tender [9]. Dysphagia is also an associated symptom when a TGDC becomes large or is located near the foramen cecum. TGDCs commonly range from 1.5-2.4 cm in size, but slightly larger cysts have been described [1,2]. In this paper, we present a case of an unusually large TGDC in an adult patient.

## Patient and observation

**Patient information:** a 44-year-old male was referred to our clinic with a 3-month history of a large anterior neck mass. His past medical and surgical history were unremarkable, and he was an active smoker with 25 pack-years. The patient was aware of the presence of a small neck mass in the last 10 years, which he reported growing in size during periods of stress. He referred no pain, neither to deglutition nor to palpation. In the last 2 weeks, he started complaining about dysphagia and mild dyspnea. He was first assessed by an endocrinologist, at the onset of his condition, who considered the lesion to be a goiter and treated the patient conservatively with levothyroxine, aspirations of the fluid, and follow-up with ultrasound (US) examination. A fine needle aspiration biopsy (FNAB) was also performed, and it was negative for malignancy.

**Figure 1:**
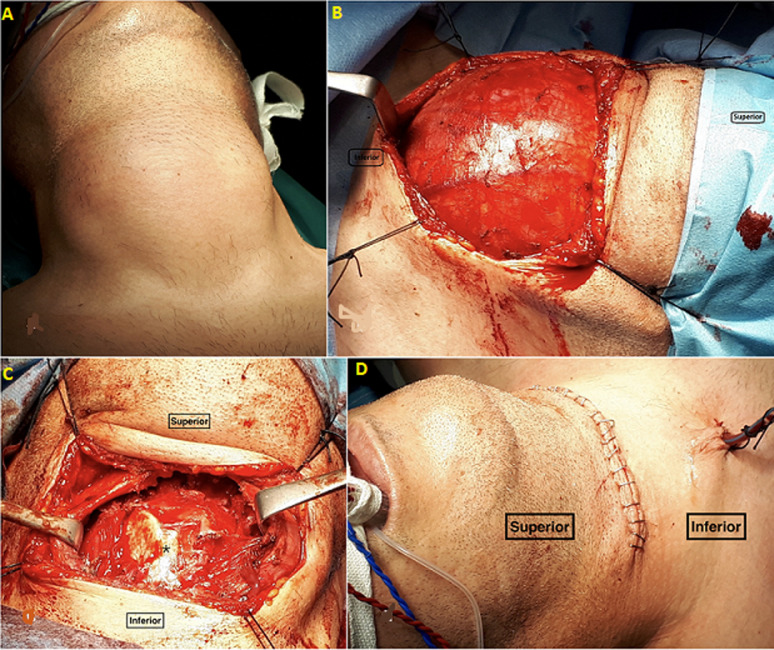
the reported large thyroglossal duct cyst: A) preoperative image showing a large thyroglossal duct cyst occupying the anterior neck space; B) intraoperative image before excision of the cyst; C) intraoperative image after excision of the cyst; the star indicates the thyroid notch; D) postoperative image

**Findings:** during our examination, no other symptoms were reported. The lesion measured around 7 cm vertically and 8 cm horizontally, was mobile, non-tender to palpation, and had a cystic consistency. It could not be assessed whether the mass moved upwards during deglutition (Figure 1A, Figure 1B, Figure 1C, Figure 1D).

**Timeline:** the lesion first appeared 10 years ago in the anterior neck, fluctuating in size. Three months ago, there was an increase of the mass in size and the patient visited an endocrinologist. A diagnosis of a goiter was made, and it was treated with levothyroxine and aspiration of the fluid. Because of the fact that the condition did not improve, the patient was referred to our clinic. Clinical examination revealed a palpable anterior neck mass, mobile, non-tender, with cystic consistency. Upper airway endoscopy and labs were beyond normal range.

**Figure 2:**
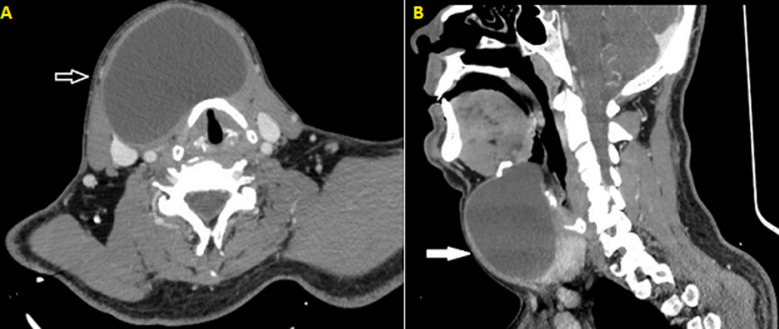
preoperative neck computed tomography of the reported case: A) axial scan; the arrow indicates the thyroglossal duct cyst measuring 8.7 cm axially and 6.6 cm anteroposteriorly; B) sagittal scan; the arrow indicates the cyst extending throughout the anterior neck space just below the hyoid bone

**Diagnostic assessment:** the rest of the clinical examination, upper airway endoscopy, and labs were beyond normal range. A computed tomography (CT) scan was conducted, and it revealed a circumscribed cystic lesion extending from the hyoid bone to the thyroid isthmus, measuring 8.7 cm axially, 6.6 cm anteroposteriorly, and 6.4 cm craniocaudally, that did not exhibit contrast enhancement (Figure 2A, Figure 2B). A clinical diagnosis of a TGDC was made based on history, clinical and radiographic findings.

**Therapeutic interventions:** the patient was treated surgically with Sistrunk´s procedure (Figure 1B, Figure 1C, Figure 1D).

**Follow-up and outcome:** the cyst was removed uneventfully, and the specimen was sent for histopathology, which confirmed the diagnosis. On a 1-year follow-up, no signs of recurrence or fistula formation were noted.

**Patient perspective:** the patient was satisfied with the successful outcome of surgery.

**Informed consent:** written informed consent was obtained from the patient for publication of this case report and of the accompanying images.

## Discussion

Thyroglossal duct cysts present in the anterior surface of the neck as a result of the failure of the thyroglossal duct to involute during embryonic life [3]. They are commonly located close to and inferior to the hyoid bone. Thyroglossal duct cysts usually present as painless neck masses, although infection of the cyst could make them tender. Their mean size is 1.5-2.4 cm in its maximal diameter but slightly bigger cysts are not infrequent, especially in adults [1,2]. We presented here the case of an extraordinarily large TGDC in a male adult, treated successfully with Sistrunk´s procedure. The number of literature reports regarding unusually large TGDCs is 10 to our knowledge (Table 1). Most of those cases had symptoms such as dysphagia, hoarseness [10], stridor, mild pain, drooling, and discomfort. None of them presented with significant dyspnea or airway obstruction, just as in our case. One patient with schizophrenia presented with a huge TGDC measuring 30x24 cm but he didn´t report dyspnea either [11]. Another case with a large TGDC, eroding the thyroid cartilage and invading the pre-epiglottic space was reported by Shaari *et al*. Even though hoarseness was present in this patient, dyspnea was absent as well [12]. A rare case of a large TGDC in a neonate was reported by Fang *et al*. Despite the cyst measuring 5x4.5x2.5 cm, which is not an extremely large size for an adult, it resulted in airway compression and breathing obstruction in the neonate [13].

**Table 1:** characteristics of other case reports of unusually large thyroglossal duct cysts

Author	Age/gender	Size (cm)	Symptoms	Procedure	Recurrence	Follow-up
Keynes, 1960	48/M	N/A	Hoarseness	Simple excision of the cyst + temporary laryngotomy	No	18 months
Shaari *et al*. 1994	44/M	7x7x2	Dysphagia, hoarseness, dryness of throat	Sistrunk’s procedure	N/A	N/A
Baisakhiya, 2011	65/M	11x2	None	Sistrunk’s procedure	N/A	N/A
McNamara *et al*., 2011	85/F	8.2x7.2x6	Dysphagia, stridor	Sistrunk’s procedure	No	N/A
Marom *et al*. 2012	35/M	30x24	Dysphagia, discomfort, drooling	Sistrunk’s procedure	N/A	N/A
Ramalingam chugh, 2013	59/M	8x6x5	None	Sistrunk’s procedure	No	6 months
Alavi garabaghi, 2015	55/M	7x7 (mediastinal TGDC)	Dry cough, retrosternal pain	Right thoracotomy excision of the cyst	N/A	N/A
El-Ayman *et al*. 2018	85/M	9.2x7.6	Discomfort with head turning	Sistrunk’s procedure	N/A	N/A
Abebe *et al*. 2019	19/F	12 in biggest diameter	Mild pain	Simple excision of the cyst	No	6 months
Mortaja et al. 2020	36/F	7.5x7x5	Dysphagia	Sistrunk’s procedure	No	N/A

M: male, F: female, N/A: not Available

A point that should be underlined is that the large size of the lesion in our patient increased the list of the potential diagnoses and led to a diagnostic error. Namely, the lesion was considered to be a goiter and was treated conservatively with levothyroxine, and aspirations of the fluid. Differential diagnosis of TGDC includes dermoid and epidermoid cysts, lymphatic malformations, branchial cleft cysts, goiter, pyriform sinus fistulas, abscesses, thymic cysts, laryngocele, primary tumors, or lymph node metastasis [3,9,14]. Thyroglossal duct cyst carcinoma may form in about 1% of TGDC, and it is mainly papillary carcinoma [15]. Thyroglossal duct cyst carcinomas range from 1-5.5 cm in size, but a case of a large TGDC carcinoma measuring 1.3x6x4.9 has been reported [16]. Thyroglossal duct cysts appear in CT as thin-walled hypoattenuating masses, whereas TGDC carcinomas exhibit enhanced wall nodularity, calcifications, and solid components within the cyst. The presence of calcifications is a more specific component for carcinoma than solid debris as the latter is present in infected TGDCs as well [17]. Fine needle aspiration biopsy could be performed but has a reported sensitivity of 56-62%, so it does not rule out malignancy, especially in less experienced hands. Thyroglossal duct cyst carcinomas frequently require additional operations besides Sistrunk´s procedure, such as total thyroidectomy or lymph node dissection, and as a result, preoperative diagnosis of malignancy could spare a second procedure [15]. Our case was initially treated with aspiration of the cyst´s fluid. This practice could shrink the size of the cyst, alleviating the patient´s signs and symptoms. However, the results are sustained just for a few days. Moreover, incision and drainage of an infected TGDC has been associated with fistula formation, even though the evidence is controversial [18]. This condition could be managed by removing skin and soft tissues around the fistula tract alongside Sistrunk´s procedure.

In our case, the TGDC was finally excised surgically with Sistrunk´s procedure. This surgical approach involves en bloc excision of the cyst, the central portion of the hyoid bone, and a core of tongue musculature up to the foramen cecum. This procedure aims to remove the entire thyroglossal tract and has shown recurrence rates between 2-13% compared to almost 50% recurrence with simple excision of the cyst [6,19]. Maddalozzo *et al*. in 2010, described the posterior hyoid space, a previously undescribed anatomic space. Its boundaries are the posterior aspect of the hyoid bone anteriorly, the thyrohyoid membrane posteriorly, the thyrohyoid membrane, and its insertion on the superior edge of the hyoid superiorly, the inferior edge of the hyoid inferiorly, and the lateral aspect of the hyoid bone laterally. Identification of the posterior hyoid space and removal of its contents are advocated by the author, since microscopic rami of the thyroglossal duct have been found in cadaveric studies. Maddalozzo *et al*. reported recurrence rates <2% with this practice [6]. One last concern is fluid spillage during surgery. In theory, the cyst should be removed intact as the definite diagnosis is made with histopathologic examination of the specimen and thus malignancy cannot be excluded. However, due to the large size of such cysts, rupture of its wall is a frequent event, as in our case. Meticulous cleaning of the surgical field with hydrogen peroxide and natural saline was applied. A larger incision could spare the cyst´s rupture, increasing the risk of injury to nerves or vessels and cosmetic defect on the other hand. Recurrence of TGDCs occurs mainly in the first year after surgical treatment [20]. In our case, 1 year after surgery no recurrence was noted. No recurrence was reported in the other cases of large TGDCs presented in Table 1 either, even though the exact length of follow-up was reported in 3 patients only.

## Conclusion

Thyroglossal duct cysts are generally well-defined lesions of the anterior neck. They usually range between 1.5-2.4 cm in diameter, although much larger cysts have been reported. Larger cysts are not linked with severe symptomatology. However, the larger size of the lesion may increase the list of potential diagnoses and lead to diagnostic dilemmas. Every effort should be made to rule out malignancy before surgery. Sistrunk’s procedure with dissection of the posterior hyoid space should be the standard of care in such cases.
